# Sexual Affectivity in Autism Spectrum Disorder: Bibliometric Profile of Scientific Production

**DOI:** 10.1007/s10508-024-02996-1

**Published:** 2024-09-13

**Authors:** Jordi Torralbas-Ortega, Victòria Valls-Ibáñez, Judith Roca, Carme Campoy-Guerrero, Meritxell Sastre-Rus, Judith García-Expósito

**Affiliations:** 1 Hospital de La Santa Creu i Sant Pau, Barcelona, Spain; 2 Nursing Care Research Group, Sant Pau Biomedical Research Institute (IIB Sant Pau), Barcelona, Spain; 3 Health Center La Serra. Catalan Health Institute, Sabadell, Spain; 4 Department of Nursing and Physiotherapy, University of Lleida, Montserrat Roig St. 25198, Lleida, Spain; 5 Nursing, Sustainability and Innovation Research Group (GREISI), Health Education, Lleida, Spain; 6 African Societies Study Group, Lleida, Spain; 7 Bachelor of Nursing, University of Andorra, Sant Julià de Lòria, Andorra

**Keywords:** Bibliometrics, Autism spectrum disorder, Sexual behavior, Sexuality, Affective symptoms

## Abstract

The aim of the present study was to describe the scientific production on sexuality and affectivity of autistic people. The inclusion criteria were articles published in all languages from the year 2000 to 2023, excluding reviews, proceedings, and other works not considered original. The search was performed in the Web of Science Core Collection and RStudio was utilized to analyze the records, with the “Bibliometrix 4.1.0” package and the VOSviewer software. A total of 314 articles were included, from the USA, Australia, and parts of Europe. The production peak was found in the year 2020, the most cited articles referred to the children’s population, and the most important journals were specialized on the subject. As for the thematic content, 29 keywords emerged that were grouped into three clusters. In the first group, children associated with vulnerability and victimization were underlined, in which multifocal interventions were needed to prevent risk; in the second, we found adolescents and the need for sex education that is adapted and comprehensive; and lastly, adults who must be able to perform an adequate transition that eases the adaptation of neurodivergent individuals.

**Supplementary Information:**

The online version contains supplementary material available at 10.1007/s10508-024-02996-1.

## Introduction

Autism spectrum disorder (ASD) is a prevailing neurodevelopmental condition that affects 1.23% of the child and youth population, and its incidence has increased in the past few years, although its estimation worldwide is non-homogeneous (Li et al., 2022; Pérez-Crespo et al., 2019). This disorder is characterized by difficulties in communication and social interaction, sensorial-perception alterations, and restricted and repetitive interests (Hervas & Pont, 2020). Thus, autistic people can experience problems in romantic relationships and affective-sexual functioning due to inappropriate behaviors, given their difficulty in understanding the physiological and psychological changes that occur during adolescence, the absence of adequate sex education, the severity of their symptoms, or the associated comorbidity (Torralbas-Ortega et al., 2022). The term affective-sexual is used to describe the intersection of sexuality and human affectivity, recognizing that the physical and emotional dimension are linked to attraction and relationships between people. The complexity and multidimensionality of human relationships is thus emphasized. The sexual and emotional aspects are closely linked. Therefore, sexual experience or behavior can increase or decrease affect (emotional component of sexual health) positively or negatively for psychosocial or physiological reasons (Wesche et al., 2019).

Despite the high prevalence of ASD, and the importance of sexuality for health, there are gaps in the literature about sexual affectivity in people with ASD (Turner et al., 2017). Some studies (Bush et al., 2021; Joyal et al., 2021; Pecora et al., 2020; Sala et al., 2020) suggest that adolescents and adults with ASD want to have sexual-affective relationships, and have sexual behaviors that are typical in most of the youth. For all this desire, the difficulties faced by people with autism centered on communication and socialization deficits, social innocence, rigidity of thought, experiences of victimization and their comorbidities, make access to satisfactory emotional and sexual experiences complicated (Dewinter et al., 2016; Hervas & Pont, 2020). Another relevant element is that autistic people have atypical tactile sensory processing, which makes it essential to identify and manage sensory needs in romantic relationships and sexual experiences (Sala et al., 2023). This need for knowledge about romantic relationships and the aspects that are part of a good relationship and privacy is expressed by adolescents with ASD in the study by Joyal et al. (2021).

Therefore, youth with ASD need special attention and care during adolescence to achieve an adequate development of their socialization and affective-sexual health (Chan & John, 2012). The sexual experiences described in the literature (Dewinter et al., 2016) and the developmental differences show the need for early and comprehensive sexual education and communication to improve emotional aspects of sexual behavior. Nevertheless, publications in the scientific literature on sexuality and affective relations of autistic people are scarce. They tend to greatly focus on the difficulties observed, rather than on preventive measures that can be implemented to facilitate a beneficial experience in affective relations (Maggio et al., 2022). In these interventions, multidisciplinary work must be done with health and education professionals, to not only adapt the content, but also the manner in which to transmit the knowledge in a way that a person with ASD can internalize it (Torralbas-Ortega et al., 2023). This education would have to address issues of sexual health and safety and also the development of skills for social relationships and courtship modeling (Cheak-Zamora et al., 2019). The family is without a doubt a key element in this process, as in sex education, parental communication allows for the development of a healthy sex life, and can also prevent negative situations (André et al., 2020). The systematic review published by André et al. concluded by affirming that communication about sex between parents and children was relevant for the transmission of information associated with values, and for favoring the development of skills to make decisions about sex, for people with ASD, just like everyone else. However, a dialogue between caregivers and youth with ASD about needs, concerns and interests can be complicated, and therefore, communication patterns must be improved (Teti et al., 2019).

With respect to the methodology used in the present study, it must be detailed that bibliometric studies allow us to map the literature, complement other types of reviews, and to assess scientific production (Zupic & Čater, 2014). There are many bibliometric studies on autism, and these examine aspects such as tele-healthcare and autism, epidemiology, intellectual disability, mobile and wearable technologies, and the role of microbiota, epilepsy, and genetics.

Considering the lack of bibliometric studies published on sexual affectivity of people with ASD, a study on the subject would allow us to identify research trends, and in line with other studies published on autism (Carmona-Serrano et al., 2020), its aim is exploratory. These studies must address external characteristics such as publications, country, authors, and journals and internal characteristics such as keywords and research foci (Xiao et al., 2021). In addition, it allows us to assess both the quantity and quality of the scientific production in this specific field (Zamit et al., 2022). Thus, it would increase our knowledge on the affective and sexual experience of people with autism, which must be broadly studied given its impact on the quality of life of individuals and their families (Maggio et al., 2022).

Given the above, the general aim of the study was to describe the scientific production on sexuality and affectivity of autistic people. The specific objectives were: (1) to identify the evolution of the scientific production with respect to aspects such as authors and their affiliations, citations, journals, languages, and countries, (2) list the scientific collaborations, and (3) specify the research elements through an analysis of the study areas and keywords.

## Method

A theoretical study is presented, which used a descriptive-retrospective bibliometric design to select, organize, and analyze documents (Lorenzo et al., 2020; Montero & León, 2007). Bibliometric studies provide insights into the patterns and impact of research literature within a particular field or across various disciplines. The methodological steps used were based on the three phases described by Fauzi (2022) and named in various studies (Selva-Pareja et al., 2023; Torné-Ruiz et al., 2023): data collection, screening, and analysis.

### Stage 1: Data Collection

#### Literature Search

To conduct the bibliometric analysis, the Web of Science Core Collection (WOSCC) database was used, as it contains a record of articles with the highest impact in the world and provides data that allow us to analyze the interconnections between the different areas of research, authors, and citations, among others. To analyze the data with Bibliometrix and VOSviewer. The keywords used for the search were: (TS = Autistic Disorder OR TS = Autism Spectrum Disorder OR TS = Autis*) AND (TS = sexual* OR TS = Affectivity). The choice of these terms is justified by: (1) the purpose of this study, and (2) the selected terms allow for an extensive exploration of the phenomenon to be investigated.

#### Identifying Relevant Studies

To identify relevant articles and include them in our study, some inclusion criteria were determined: (1) articles that were published on the subject, (2) original articles, (3) in any language, and (4) records published between January 1, 2000, and February 10, 2023. On the other hand, the exclusion criteria were articles not considered primary, scientific, or quantitative or qualitative original articles (reviews and other documents such as books, letters to the editor, editorial materials, and notes, among others). The search took place on February 10, 2023, to ensure the correct inclusion of all articles published so far

### Stage 2: Screening

####  Eligibility Criteria

The filter available in WoS “document type” was applied to determine that all articles included met the established requirements.

#### Study Selection and Data Collection

The following study variables were considered: year of publication of the articles selected, number of references (citations and self-citations), important authors in the field of interest, publication journals, areas of interest and research, country of origin and collaboration networks between them, languages, and most-utilized keywords.

This analysis carried out with the keywords through co-words and co-occurrence frequencies is based on the assumption that in a specific topic a set of signal-words that reflect a basic content can be abstracted from the literature and its frequency of co-appearance marks the relevance of the topics and their relationships (Zhang et al., 2012). To select the articles, two authors evaluated the title independently. The disagreements that could emerge were resolved by consensus with another reviewer.

### Stage 3: Analyzing the Data

#### Performance Analysis

A descriptive analysis was carried out with the relevant data provided by the WoS database such as year, number of records and count of citations on publications related to the topic.

#### Science Mapping

To analyze the data, the RStudio software program was utilized, with the R-package “Bibliometrix 4.1.0”, which provides a set of tools for quantitative research in bibliometry and scientometry (Aria & Cuccurullo, 2017; Guleria & Kaur, 2021).

In addition, the VOSviewer software was used to analyze and visualize networks of coexistence of important terms extracted from both the titles and abstracts of the scientific literature by representing keywords with different colors and circles of different sizes (Torné-Ruiz et al., 2023).

## Results

### Results from the Search in the Web of Science Core Collection Database

**Fig. 1:**
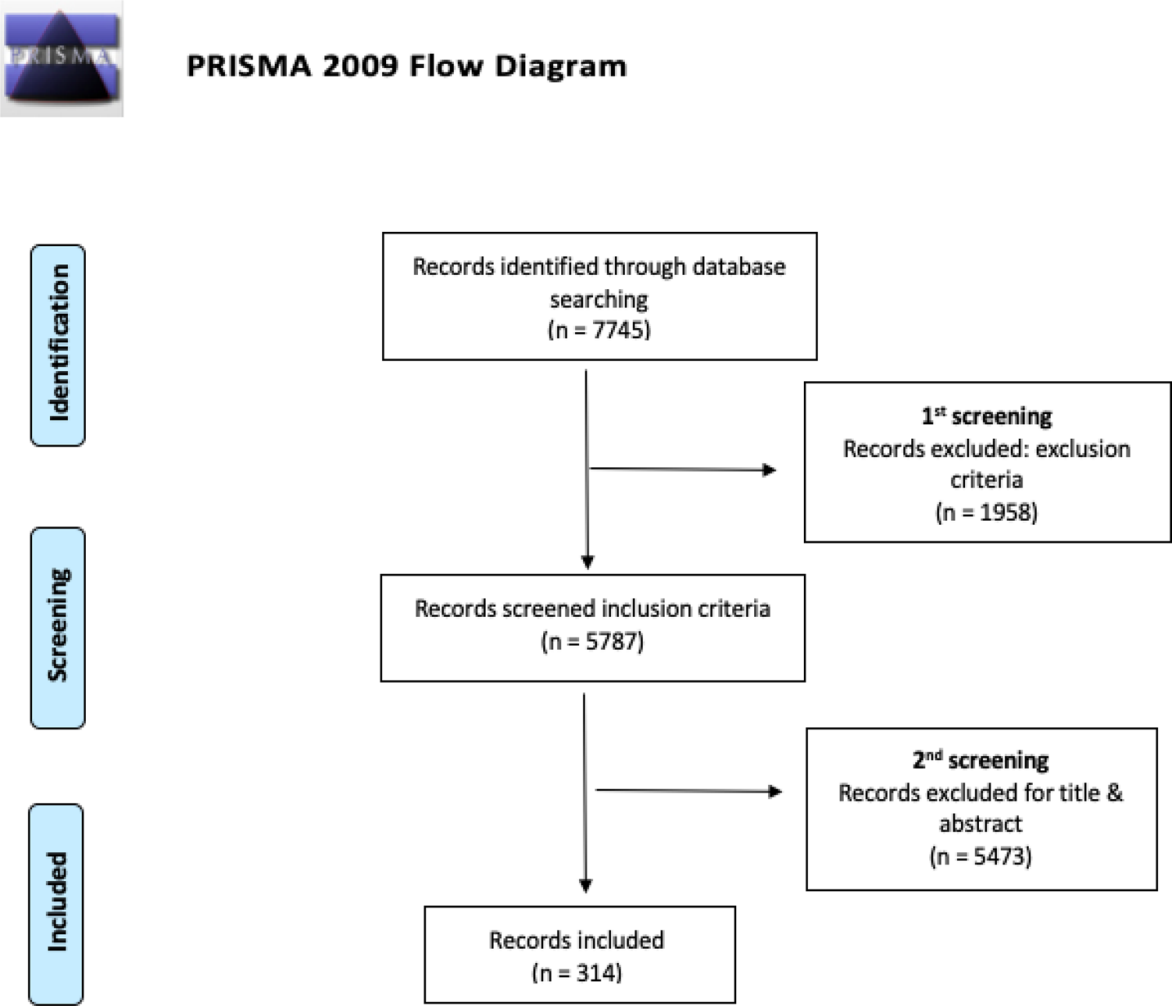
Flow diagram adapted from Preferred Reporting Items for Systematic Reviews and Meta-Analyses (PRISMA)

The search strategy utilized on the Web of Science Core Collection (WOSCC) provided a total of 7745 records, which decreased to 5787 articles after the application of the inclusion and exclusion criteria. Lastly, after the evaluation according to title, a total of 314 articles were included in the analysis. The publications removed mainly dealt with studies using animals, genetic or hormonal studies, negative effectivity or type D personality, or the work environment, which were not associated with sexual affectivity and autism in any shape or form. The entire selection process is shown in the PRISMA flow diagram (Fig. 1).

### Year of Publication and Number of Citations

**Fig. 2:**
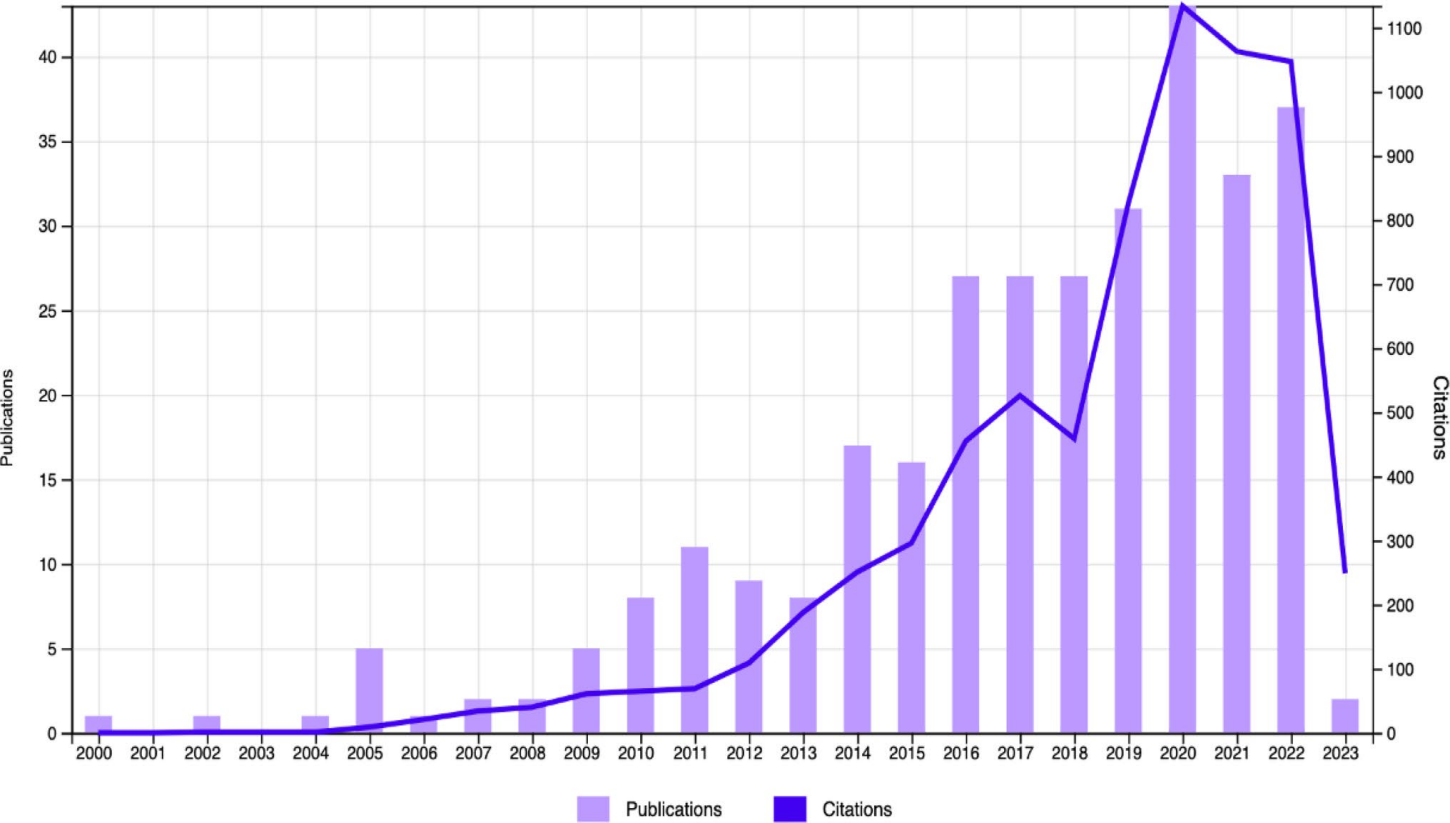
Number of publications and citations per year in WOSCC

From the year 2000 to 2013, only a total of 46 records related to the subject were found. In turn, in the last 10 years, an exponential increase in the number of publications can be observed. The year 2020 being the most productive, with a total of 43 original articles, followed by the years 2022 (37 records), and 2021 (33 records). With respect to the number of citations, the same trend was found, with an exponential growth observed through time. The studies included in the bibliometry were cited 6671 times (4941 without self-citations), with a mean of 21.25 citations (Fig. 2).

The most-cited article was published in 2016 by Bargiela et al., “The Experiences of Late-diagnosed Women with Autism Spectrum Conditions: An Investigation of the Female Autism Phenotype,” with total of 347 citations. The next 5 most-cited articles were directly related to the child population: In 2011 by Miodovnik et al., “Endocrine disruptors and childhood social impairment”; in 2006 by Konstantareas et al., “Affect regulation and temperament in children with autism spectrum disorder”; in 2011 by Billstedt et al., “Aspects of quality of life in adults diagnosed with autism in childhood A population-based study”; in 2005 by Mandell et al., “The prevalence and correlates of abuse among children with autism served in comprehensive community-based mental health settings” and in 2016 by Holt et al., “Young people with features of gender dysphoria: Demographics and associated difficulties.” All of them obtained a total of 876 citations as a set.

### Authors and Affiliations

As for the authors, the five most prolific ones were Stokes with 12 articles (3.82%), Holmens with 11 (3.50%), Himle with eight (2.55%), Greaves-Lord with seven (2.23%) and Nichols with six (1.91%) (Supplementary Information_Supplementary File 1). Likewise, the author with the most publications, Stokes had the greatest trajectory (2005 a 2020), and also obtained the highest number of citations, for a total of 97 citations. Few authors show an increase in their individual production over time, with a tendency to decrease in many of the cases studied. As for the affiliations, the five most important ones, according to published records were: Deakin University (Australia) (*n* = 26), University of Utah (USA) (*n* = 22), Vanderbilt University (USA) (*n* = 13), University of Gothenburg (Sweden) (*n* = 12), and Ohio State University (USA) (*n* = 11). These have maintained an increasing scientific production in recent years (Supplementary Information_Supplementary File 2).

### Journals and Areas of Research

A total of 160 journals published on ASD and sexuality or affectivity, but the five journals with the most publications were: Journal of Autism and Developmental Disorders (*n* = 42), Sexuality and Disability (*n* = 31), Autism (*n* = 16), Autism in Adulthood (*n* = 9), and Research in Autism Spectrum Disorders (*n* = 8). Table 1 presents the main metric characteristics of these journals as a result of their indexing in the database.

**Table 1:** Characteristics of the journals with the most relevance in different classifications

Journal	JIF* (2022)	Subject area and category	Edition	Rank category	JIF quartile
Journal of Autism and Developmental Disorders	4.35	Developmental Psychology	SSCI*	20/78	Q2
Sexuality and Disability	1.90	Rehabilitation	SSCI	42/73	Q3
Autism	6.68	Developmental Psychology	SSCI	6/78	Q1
Autism in Adulthood	N/A	Developmental Psychology	ESCI*	N/A	N/A
		Rehabilitation			
Research in Autism Spectrum Disorders	3.29	Special Education	SSCI	4/44	Q1
		Psychiatry	SSCI	76/143	Q3
		Developmental Psychology	SSCI	32/78	Q2
		Rehabilitation	SSCI	9/73	Q1
Archives of Sexual Behavior	3.8	Clinical Psychology	SSCI	38/131	Q2
		Interdisciplinary Social Sciences	SSCI	15/110	Q1

^*^Journal impact factor by Clarivate; SSCI: Social Sciences Citation Index; ESCI: Emerging Source Citation Index

**Fig. 3:**
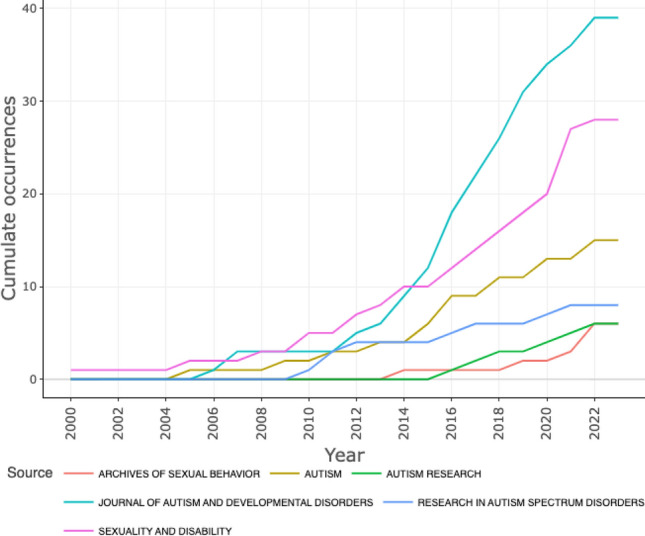
Sources’ Production over Time

This production greatly increased in the last five years in most of the journals, with an exponential growth in two of them (Fig. 3). Among the journals that have dealt most with the relevance of the topic with sustained increase has been Journal of Autism Developmental Disorders.

A total of 48 different areas of research were found related to the subject. Among the five that published the most on ASD and sexual affectivity, we found: Developmental Psychology (*n* = 108, 34.39%), Rehabilitation (*n* = 79, 25.16%), Psychiatry (*n* = 56, 17.83%), Special Education (*n* = 38, 12.10%), and Clinical Psychology (*n* = 26, 8.28%).

### Countries and Publication Language

A total of 39 countries published on the subject, but the ones with the highest production were: the USA with 140 documents (44.59%), UK, with 44 (14.01%), and Australia with 28 (8.92%). The most cited country coincided with the one with the most publications, for a total of 2741 citations (2403 without self-citations), and the year with the most citations was 2022, with a total of 413.

**Fig. 4:**
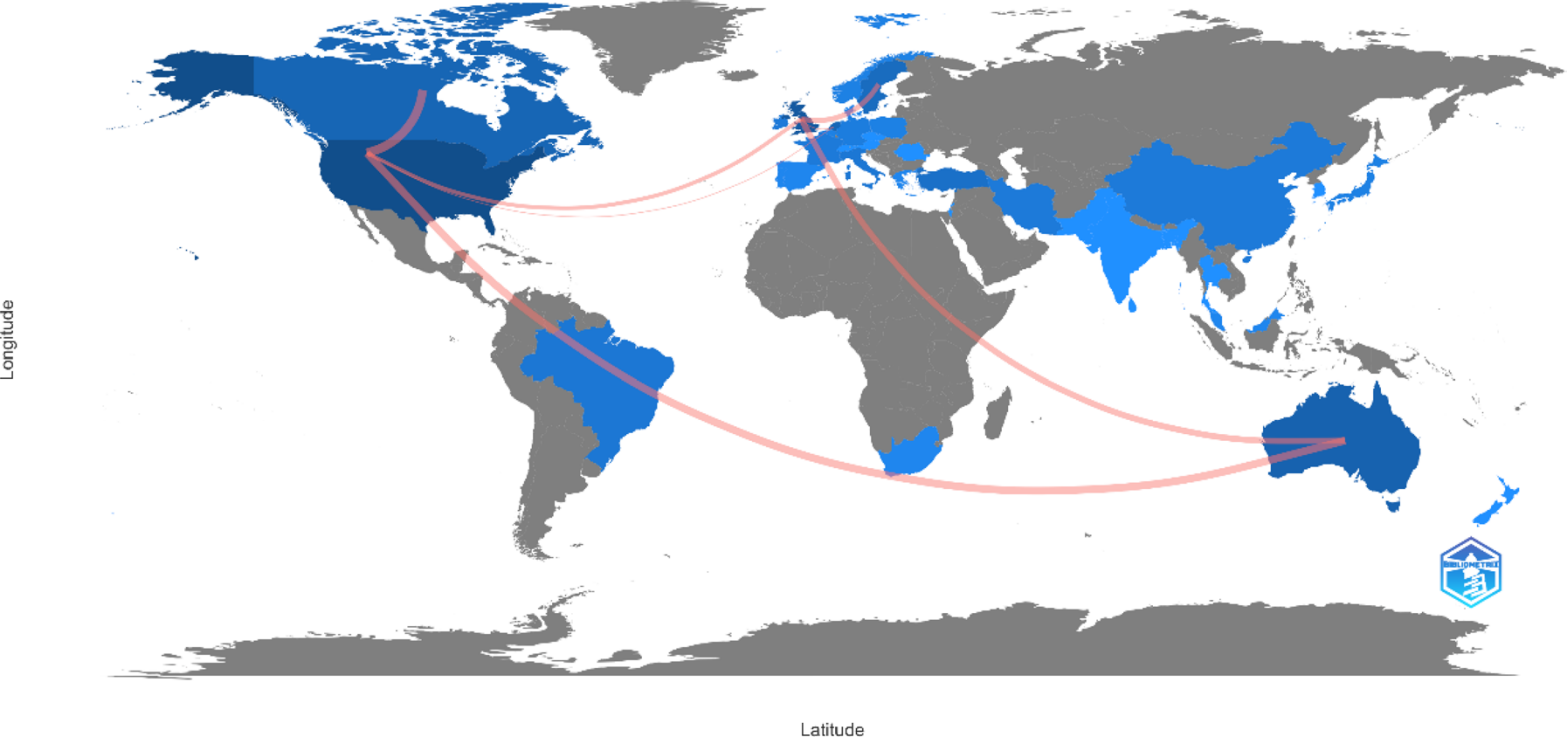
Countries with the most scientific production on ASD and sexuality or affectivity. *The intensity of the blue color indicates a greater contribution to scientific production. The gray color indicates no scientific production about the subject. The red lines indicate collaboration between countries

As for the number of collaborations established between the different countries, it was observed that the greatest number of collaborations coincided with the countries that had published the most on the subject. These were: USA, Canada, Australia, UK and Sweden (Fig. 4).

The articles selected for the analysis were written in 6 languages, with the most utilized being English, in 97.13% of the articles (*n* = 305), followed by French, in four articles, and Portuguese, in two. Lastly, there was only one article each in Spanish, German, and Turkish.

## Keywords and Clusters

With respect to the keywords, considering the Keywords Plus, the 5 most-utilized words were: children (*n* = 114), adolescents (*n* = 98), adults (*n* = 93), individuals (*n* = 50) and high-functioning autism (*n* = 46).

**Fig. 5:**
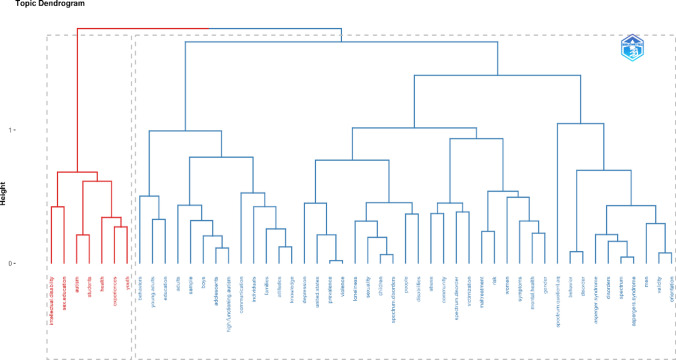
Topic dendrogram of MCA of Keyword Plus

A multiple correspondence analysis (MCA) of the Keywords Plus with the greatest occurrence showed two differentiated clusters. The MCA is a conceptual structure that groups common concepts by K-means for the creation of clusters (Aria & Cuccurullo, 2017). On the one hand, we find the most concise (red color), highlighting keywords such as intellectual disability, sex education, autism, students, health, experiences and youth. And on the other, we find the other most extensive cluster (blue color), which included words such as communication, depression, loneliness, spectrum disorders and behaviors (Fig. 5). Therefore, MCA involves examining the relationships and patterns between different keywords or assigned terms in order to discover structures or associations in the data. Thus, these can be represented graphically with a dendrogram. On the vertical axis, the distance or dissimilarity between the elements through height and bifurcations is shown, and on the horizontal axis the groupings or bifurcations between elements.

**Fig. 6:**
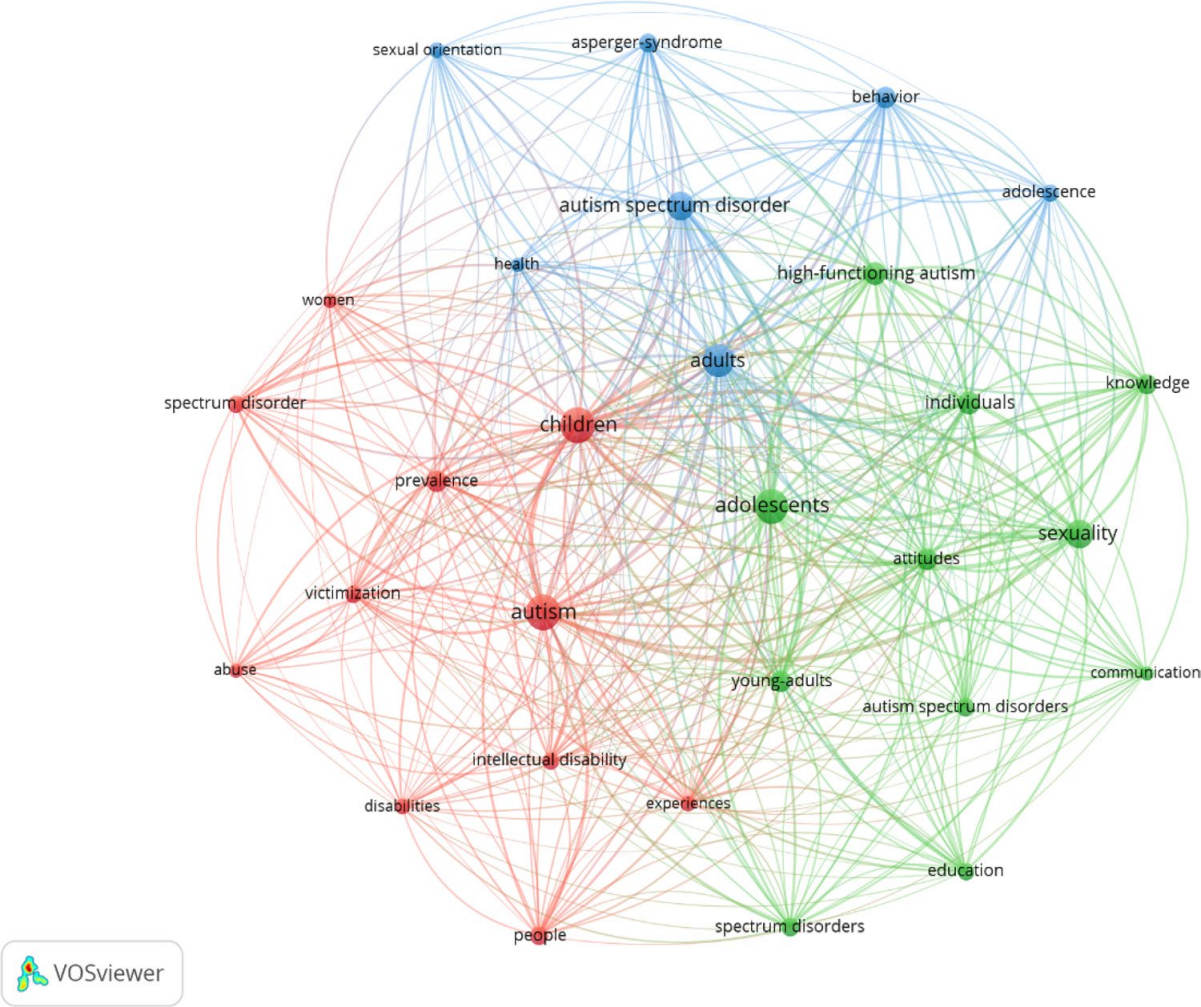
Network map of the 29 keywords with a frequency of more than 20 occurrences

When analyzing the co-occurrence, the method utilized to reveal the thematic content of the set of publications was based on all the keywords: the author’s keywords, and the Keywords Plus (assigned by the WOS database program). Clusters or keyword networks are created by co-occurrence analysis (Aria & Cuccurullo, 2017). All the keywords with a minimum of 20 occurrences were selected, and a total of 29 terms were identified. The size of the nodes is proportional to the number of articles with a specific keyword, and those with a greater rate of coincidence tended to be close to each other (Marx et al., 2017). In the resulting map, it was observed that six of the words with the greatest occurrence were: autism (117 times), children (117), adolescents (106), adults (100), autism spectrum disorder (77) and sexuality (76) (Fig. 6). Therefore, it can be observed that the size of the node correlates positively with the frequency, that is, the node is larger if the word has a higher frequency. The terms autism, autism spectrum disorder and sexuality were the most utilized by the search strategy in the WOSCC database, so they are not taken into account in the analysis. Likewise, it was observed that many of the studies included in the analysis were centered on the children and adolescent populations, with these keywords being very common.

On the map, three clusters can be differentiated that included diverse items: (1) cluster 1 (red, eleven items): children (Highlighted word), abuse, autism, disabilities, experiences, intellectual disability, people, prevalence, spectrum disorder, victimization, women; (2) cluster 2 (green, eleven items): adolescents (Highlighted word), attitudes, autism spectrum disorder, communication, education, high-functioning autism, individuals, knowledge, sexuality, young-adults; (3) cluster 3 (blue, seven items): adults (Highlighted word), Asperger syndrome, autism spectrum disorder, behavior, health, adolescence, sexual orientation. Likewise, when delving into each of the clusters, different keyword trends were observed: cluster 1 was more directed toward children, cluster 2 was oriented toward terms centered on the adolescent population, and cluster 3 to adulthood, ASD, and sexual orientation.

Finally, Table 2 presents a quantitative analysis taking into account Links (simultaneous connection between two words), Total Link strength (total strength of the links between words) and Occurrence (frequency of appearance). Regarding the keywords with the highest Links, we can highlight Cluster 1: Autism, children, prevalence and victimization (28); in Cluster 2: Adolescents, attitudes, high-functioning autism, individuals, sexuality and young-adults (28); and in Cluster 3: Adults, autism spectrum disorder and health (28). In reference to the total link strength, the words that obtain the highest score in Cluster 1 are Children (497) and Autism (446), in Cluster 2 it is Adolescent (486), and in Cluster 3: Adults (438), coinciding with those with the highest occurrences.

**Table 2:** Keywords analysis clusters 1, 2, and 3

	Item	Links	Total link strength	Occurrences
Cluster 1	Abuse	24	101	24
	Autism	28	446	117
	Children	28	497	117
	Disabilities	24	90	20
	Experiences	26	120	21
	Intellectual disability	25	109	27
	People	26	136	31
	Prevalence	28	181	41
	Spectrum disorder	25	120	27
	Victimization	28	138	26
	Women	26	95	22
Cluster 2	Adolescents	28	486	106
	Attitudes	28	219	40
	Autism spectrum disorders	26	113	30
	Communication	24	116	20
	Education	26	134	28
	High-functioning autism	28	267	49
	Individuals	28	257	50
	knowledge	27	208	37
	Sexuality	28	358	76
	Spectrum disorders	25	126	33
	Young-adults	28	206	40
Cluster 3	Adults	28	438	100
	Asperger syndrome	26	133	31
	Autism spectrum disorder	28	303	77
	Behavior	27	218	47
	Sexual orientation	22	112	25
	Adolescence	25	126	26
	Health	28	124	23

## Discussion

### General Aspects of Bibliometric Indicators

The findings from the present review allow us to identify a positive progressive evolution of the scientific production on sexual affectivity of people with ASD, with this trend becoming sharper in the last 10 years (2013–2023), and more specifically, the peak in production was observed in the year 2020. This growth in scientific production shows scientific concern about the topic (da Silva et al., 2021). The most-cited articles referred to children. This focus on childhood could be justified, taking into account the reasons given by Hirota and King (2023) for an increase in the estimated prevalence of autism that is associated with changes in diagnostic criteria, in the performance of screening instruments and in greater public awareness.

The bibliographic indicators revealed that the most prolific journals (which publish the greatest number of articles on the topic) belonged to the three upper quartiles: Q1 (*n* = 3), Q2 (*n* = 2), and Q3 (*n* = 2). Therefore, the interest in sexuality and autism in higher quartile journals is inferred. These journals had a Journal Impact Factor (JIF) ranging between 6.68 and 1.90. The JIF is considered a measure of journal quality, although its calculation is a controversial issue (Siler & Larivière, 2022). The main journals were specialized on autism and related to areas such as Developmental Psychology, Rehabilitation, Special Education, and Psychiatry.

With respect to the authors and their affiliations, they were affiliated to universities, according to geographical areas, Australia, North America, and Europe. The production in Asian countries was more incipient, just as in the African countries. Even when observing the world map (Country Collaboration Map), it was evident that in Africa, the scientific production came mostly from South Africa, and in Asia, mostly from China, followed by other countries such as India, Turkey, and Iran. Lastly, in South America, the contributions by Brazil were underlined. This distribution by countries and regions is similar to the results obtained in the study by da Silva et al. (2021) on sexuality and adolescence.

These differences in global scientific production have an impact on global health equity (Cash-Gibson et al., 2018). Although global health conditions have positive trends, a detailed analysis per region, country or even within one nation shows an increase in inequalities in health levels (Lima-Barreto, 2017). The results of this study by region and scientific production suggest this more anthropological view, since research on autism is highly focused on high-income Western countries (de Leeuw et al., 2020). Although autism is universal, it was irregularly identified in many different regions of the world, with the African continent having the most unspecific data (Bakare & Munir, 2011). The review of Abubakar et al. (2016) concluded that prevalence studies and interventions were almost non-existent. Also, it provided very important data; firstly, the need for validated and standardized instruments in the African context toward early screening and detection, and secondly, psychosocial aspects of ASD that imply a load for African communities. Thus, more care and management of this disorder are needed.

The findings on the scientific production in Asian countries show a different situation to the African one, although the cultural and social connotations are maintained. A review study (Shorey et al., 2019) showed that raising children with ASD can result in problems of mental health, divorce, and unemployment. This overload or stress associated with raising these children is aggravated by cultural and environmental factors, given the marked traditional and conservative character of Asian societies. This demands support programs for families that are adapted to their reality, to ensure a positive experience in raising autistic children (Shorey et al., 2019). Additionally, it must be underlined that studies such as that by Sritharan and Koola (2019) showed us that cultural differences have a direct relation with the process of diagnosis, the manner of understanding the disorder, and even with blaming oneself for its presence, as well as posterior management of the person with ASD, and the search for aid resources. The findings of this study would reinforce, from a perspective of bibliometric indicators, the conclusion of the study by de Leeuw et al. (2020) on autism research with culturally and contextually appropriate methods.

Along this line, it must also be underlined that the collaboration between countries was very scarce, and when they occurred, they were centered on Western contexts (Europe, North America, and Australia). International cooperation is the engine of internationalization of research, through strategies and instruments of scientific policy of the different countries and institutions (Sebastian, 2019). However, cooperation is very complex, especially within formal and global frameworks, although they are not as complex in informal contexts, with the recognition of spontaneous collaborations in the informal context, through agencies or self-financing (Sebastian, 2019). It is also necessary to highlight that situations of crisis such as COVID-19 changed the rates of international collaborations and publications, but we must consider geopolitical and economic elements (Lee & Haupt, 2021) and examine this trend during the post-pandemic period, and when facing other health situations.

### Analysis of Clusters by High Co-occurrence Words

In the analysis of the grouping of authors’ keywords and Keyword Plus included in each cluster, a relationship between the 3 main terms was observed, one for each life stage: children, adolescent, and adult. The cluster around the node “children” (in red) included terms that mentioned childhood and danger: it was not an exclusive mention in the case of autistic children, but it was clearly present in the studies focused on their sexuality, a fact that strengthens the presence of terms such as “victimization,” “abuse,” related to “women,” and “intellectual disability/disabilities,” who show a greater vulnerability. This situation of vulnerability is shown in research where women are under-represented or men are diagnosed with autism more frequently (Nordahl, 2023). Some studies showed high rates of victimization of individuals with ASD, relating individual and contextual factors, which make them more vulnerable to abuse as victim and perpetrator (Sreckovic et al., 2014). Likewise, the legal problems derived from inadequate behaviors in the affective and sexual domain were directly associated with autism (Allely & Creaby-Attwood, 2016; Creaby-Attwood & Allely, 2017).

In the cluster related to the “adolescents” node (in green), there was a high prevalence of terms such as “education,” “communication,” or “knowledge,” and a direct relationship between adolescence and sex education was established. Different studies (Beato & Correia, 2022; Chianese et al., 2021) detailed the need to promote formal (adapted) sex education, avoiding focusing on the mechanics of sex or contraception, and more on socio-emotional skills and intimacy. The need for the specific adaptation of sex education for people with autism is based on dealing with aspects such as difficulty in communication and social thinking, the ability to understand complex information, or the sensorial experience of physical contact (Solomon et al., 2019).

Lastly, in the cluster related to the terms “adult” and “adolescence” (in blue), we found terms such as “health,” “behavior,” or “sexual orientation”. The behavior of young and adult people with autism has been studied in diverse articles, habitually utilizing cultural criteria for its assessment (Kellaher, 2015; Martinello, 2015), and normally considering situations of risk as the element of analysis. The aspects of sexual orientation and gender identity have been subjects of intense debate among researchers (Corbett et al., 2022). A considerable increase was found in the prevalence of orientations different from heterosexuality among the population of autistic people (Attanasio et al., 2022; Dewinter et al., 2017), and a greater fragmentation of sexual identity with a greater fluidity in their temporal perceptions in neurodiverse individuals (Corbett et al., 2022). Strang et al. (2021) described an intervention program centered on the needs perceived by families, the autistic people, and health professionals, to specifically affect aspects centered on the variation of gender, which could be applied in a cross-sectional manner, to facilitate the adaptation of neurodiverse people to these situations. Additionally, it is worth detailing the importance of understanding “disability and diversity” from a critical and contextualized vision of the person with ASD, the representation of autism in its pediatric condition exposes adults to infantilization that sometimes involves discriminatory practices (marginalization, social isolation, moral judgments) and a misconception of sexual behaviors (Lo Bosco, 2023).

### Limitations

This is a descriptive study on scientific production. The description has been carried out using standardized tools to facilitate the understanding of basic elements of the bibliometric study. The choice of search words can be a limiting factor. Although the search was used as a “topic”, we facilitated the inclusion of the keyword in Title, Abstract, Author Keywords and KeyWords Plus®. In subsequent studies, bibliometric analysis can be expanded with other databases, new citation analysis or more qualitative and/or semantic measurements. These elements would offer more in-depth information that complements this more initial and introductory study, and with other types of literature review studies that would expand the vision of the phenomenon under study.

### Conclusions

The growing scientific production shows an interest in this topic, highlighting publications on children and in Western countries such as the USA, Canada, Australia, the UK and Sweden. The limitation in other regions of the world, especially in the African and Asian continents, limits research and therefore, progress in more contextualized knowledge. The management of autism must consider social and cultural aspects. The keywords show a greater concurrence in the life cycle (childhood, adolescence, adulthood), and therefore the need for care adapted to the aspects of sexual affectivity and the characteristics of autistic people in the different stages of life.

## Supplementary Information

Below is the link to the electronic supplementary material.

Supplementary file1 (DOCX 93 KB)

Supplementary file2 (DOCX 55 KB)

## Data Availability

All data have been described in the manuscript.
