# Supplementary figures and images for: Sexual Affectivity in Autism Spectrum Disorder: Bibliometric Profile of Scientific Production
Source: Arch Sex Behav. 2024 Sep 13;54(2):673–84. doi: 10.1007/s10508-024-02996-1 (PMC11836160; doi:10.1007/s10508-024-02996-1)

**Figure SF1.** Authors’ Production over Time.

**
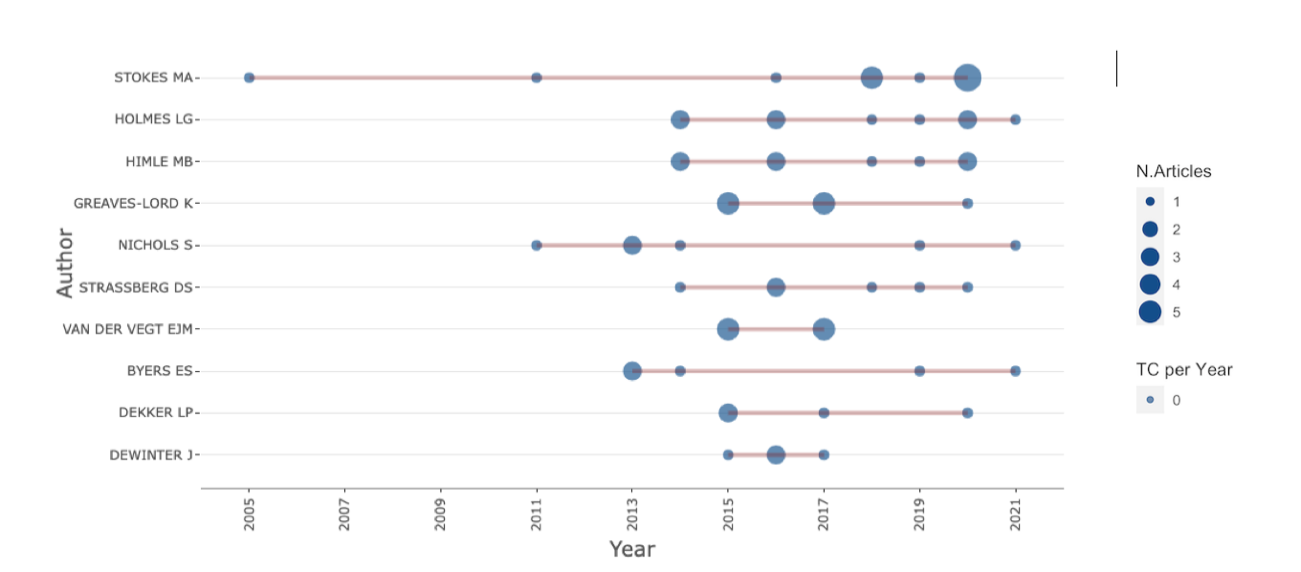
**

Supplement: Supplementary file 1 — Supplementary file1 (DOCX 93 KB) [file 10508_2024_2996_MOESM1_ESM.docx]

**Figure SF2.** Affiliations’ Production over Time.

**
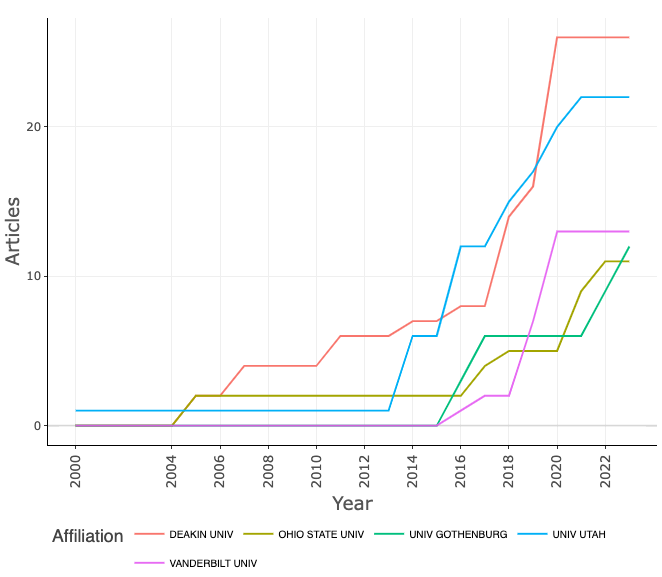
**

Supplement: Supplementary file 2 — Supplementary file2 (DOCX 55 KB) [file 10508_2024_2996_MOESM2_ESM.docx]
